# Shared and distinct patterns of cortical morphometric inverse divergence and their association with empathy in dancers and musicians

**DOI:** 10.1038/s41598-025-13416-2

**Published:** 2025-08-05

**Authors:** Yuanyuan Yu, Hui He, Ruikun Yang, Lan Yang, Yayun Liu, Dezhong Yao, Cheng Luo, Frank Polick, María Luisa Bringas Vega, Benjamin Klugah-Brown, Jing Lu, Qiushui Xie, Lupeng Yue, Mingjun Duan, Gujing Li

**Affiliations:** 1 The Clinical Hospital of Chengdu Brain Science Institute, MOE Key Laboratory for Neuroinformation, School of Life Science and Technology, University of Electronic Science and Technology of China, Chengdu, China; 2 The Clinical Hospital of Chengdu Brain Science Institute, Department of Psychiatry, Chengdu Mental Health Centre, The Fourth People’s Hospital of Chengdu, Chengdu, China; 3 School of Psychology and Neuroscience, University of Glasgow, Glasgow, UK; 4 Cuban Neuroscience Center, La Habana, Cuba; 5 China-Cuba Belt and Road Joint Laboratory on Neurotechnology and Brain-Apparatus Communication, University of Electronic Science and Technology of China, Chengdu, China; 6 Beijing Dancing Academy, Beijing, China; 7 Education Center for Students Cultural Qualities, University of Electronic Science and Technology of China, Chengdu, China

**Keywords:** Dancer, Musician, Brain plasticity, Brain similarity, Morphometric inverse divergence, Human behaviour, Cognitive neuroscience

## Abstract

Neuroimaging research has demonstrated that long-term dance and music training can induce structural changes in the brain. However, most previous studies have focused on isolated structural metrics, neglecting the interregional similarities across cortical areas. In this study, we applied a novel morphometric measure, Morphometric Inverse Divergence (MIND), to assess cortical structural similarity in individuals with professional dance or music training. A total of 89 participants were incorporated in the study, including 25 dancers, 24 musicians, and 40 healthy controls (HC). Our findings showed that both dancers and musicians exhibited higher MIND values compared to HC, with musicians displaying significant differences particularly in the default mode network and somatomotor network. Furthermore, MIND values between the insula and superior parietal lobule, as well as between the superior frontal gyrus and cingulate gyrus, were positively correlated with the total IRI score and the Fantasy subscale. Both shared and distinct MIND patterns were identified between dancers and musicians. Musicians exhibited greater structural similarity in auditory cortical regions, whereas dancers showed increased similarity in visual and kinesthetic areas. Importantly, only the dancer group demonstrated a significant association between MIND values and empathic imagination. These findings provide new insights into how long-term artistic training influences cortical structure and social cognitive abilities.

**Supplementary Information:**

The online version contains supplementary material available at 10.1038/s41598-025-13416-2.

## Introduction

Dance and music are two closely related and highly skilled art forms, both requiring extensive practice to achieve professional mastery. While they share several similarities, they also have distinct differences in artistic performance and emotional expression^1–4^. Both dance and music involve rhythm and beats and are capable of conveying emotional message. They transcend cultural and linguistic barriers, providing a universal means of emotional communication^5–8^. Music communicates through sound and subtle movement, primarily engaging auditory and motor interactions proces^9–12^. In contrast, dance expresses itself through large-scale of whole body movement and spatial movement, engaging constant observation and simulation with synchronization of musical beats. Dance practice process thus fully engages both visual-auditory integration and kinaesthetic sensations^13–17^. These differences in modality and training may lead to varying effects on emotional perception and expression.

Empathy, the ability to understand and share the emotions of others^18^is a key emotional response in both dance and music. Both art forms incorporate a range of emotional expressions, and both dance and music training have also been shown to enhance empathy in healthy individuals^19–20^. Dance and music therapy are also widely used to improve emotion regulation and foster empathy in individuals with mental disorders, including schizophrenia^21–23^anxiety and autism^24]– [25^. The distinct characteristics of dance and music may contribute to the different mechanism underlying empathy. Specifically, dance may enhance kinesthetic empathy through extensive movement simulations, which improve the ability to understand the intentions of others^26–29^. Kinesthesia, the awareness of one’s body position and movement derived from proprioceptive signals from muscles and joints, serves as a foundational element in dance^30^. Beyond guiding dance, kinesthetic perception is also intimately linked to the mechanisms underlying empathy. Thus, kinesthetic empathy specifically describes the capacity to emotionally connect with and interpret people’s internal state by perceiving their movements, posture, and bodily expressions^31^. Study have shown that synchronization expert dancers like tango dancers have showed higher kinesthetic empathy^32^. Music, on the other hand, fosters empathy by stimulating emotional resonance within musical compositions^33,34^. Research has found that people who listening to sad music under high empathy condition facilitated the emotion of nostalgia and facilitated the emotion of power in happy content^35^and other study found that individuals with higher empathy behave better when performing music with others^36^. These differences in how dance and music training engage empathy likely arise from the unique focuses of each discipline.

Prolonged, domain-specific practice induces measurable functional and structural neuroplasticity across a range of expert populations. Visual artists, for example, show strengthened functional connectivity within occipito-parietal circuits that support visuospatial integration and mental imagery^37^. Chess masters display augmented coupling in the default-mode and fronto-parietal networks that subserve strategic reasoning^38^. Olympic-level rowers and swimmers recruit partially divergent networks during higher-order cognitive tasks^39^underscoring discipline-specific adaptations. On the structural side, elite soccer players exhibit increased white-matter integrity in the bilateral acoustic and superior thalamic radiations, together with greater microstructural coherence of the corpus callosum^40^while gymnasts demonstrate enlarged gray matter volume in the post-central gyrus and inferior parietal lobule^41,42^.

Dance and music training—skills that likewise demand long-term, multimodal practice—produce convergent adaptations in sensorimotor, auditory, visuospatial and affective networks^43,44^. Moreover, these training-induced plasticity in dance and music may also related to enhanced empathy. In musicians, empathy traits have been associated with increased activation in the right ventrolateral prefrontal cortex, a region implicated in emotional processing^45^. Wallmark et al. further demonstrated that both affective and cognitive components of empathy modulate activity in the supplementary motor area, inferior frontal gyrus, insula, and temporoparietal junction—key regions involved in embodied simulation and mentalizing^46^. Among dancers, Wu et al. reported significantly higher empathic concern scores relative to non-dancers, with gray matter volume in the subgenual anterior cingulate cortex positively correlating with empathy^47^. Our previous work was the first to identify convergent patterns of insular connectivity associated with empathy across both musicians and dancers^48^. Collectively, these findings suggest that long-term artistic training not only reshapes the structural and functional architecture of the brain, but may also facilitate the development of complex social-cognitive abilities.

Despite growing interest in the neural basis of empathy, few studies have examined how long-term training in dance or music shapes the underlying brain architecture. Existing research has primarily focused on isolated structural or functional markers, often overlooking the complex, large-scale connectivity patterns that may support empathic processing. Notably, no prior studies have investigated changes in regional structural similarity induced by dance or music training using similarity-based connectomic approaches. Sebenius et al. introduced Morphometric Inverse Network Divergence (MIND), a novel framework that preserves the full multivariate distribution of voxelwise morphometric features within each region^49^. This approach enables a more nuanced characterization of cortical organization and enhances sensitivity to interindividual differences driven by developmental and genetic variation, which have been widely employed to detect similarity in schizophrenia^50^ and depression patients^51^. In the present study, we used MIND to explore the effects of dance and music training on the brain’s empathy network from a whole-brain perspective. We conducted a comparative analysis of the cortical similarity patterns in dancers, musicians, and healthy controls, with a particular focus on their association with empathy. We hypothesize that (1) dancers and musicians will exhibit both shared and distinct MIND pattern compared to healthy control; and (2) these differential brain patterns may be associated with their respective levels of empathy.

## Methods

### Participant information

A total of 89 participants were included in this study: 25 professional dancers, and 24 professional musicians, and 40 healthy control(HC). The expert groups comprised college students majoring in modern dance and Western string instruments from Southwest Minzu University and the University of Electronic Science and Technology of China. Before the formal experiment, senior dancers and musicians assessed the proficiency of the experts. Specifically, dancers were evaluated through three tasks: (1) a flexibility task, (2) an imitation task, and (3) an improvisation task. Musicians were assessed with two tasks: (1) a continuous performance task and (2) a sight-reading task. Additionally, we examined the participants’ training history, excluding those with irregular training periods. Experts who met the criteria were included in the analysis. In contrast, the control group had no formal training in dance or music. The dancers and musicians had an average of over seven years of training, with an average of 12 h of practice per week. All participants were right-handed, as determined by the Edinburgh Handedness Inventory^52^. For further details on the experimental design, please refer to our previous research^48^.

Our previous research has confirmed structure and function coupling differences between dancers, musicians and HC^53^.In this study, we specifically investigate the structural similarity and difference among these three groups. The study was registered in the Chinese Clinical Trial Registry (ChiCTR2200059526) and received approval from the local Ethics Committee of the University of Electronic Science and Technology of China. All participants provided written informed consent and the study adhered to the ethical principles outlined in the 1964 Declaration of Helsinki^54^.

### Behavioral data

Empathy was assessed using the Chinese version of the Interpersonal Reactivity Index (IRI-C)^55,56^, which evaluates both cognitive and emotional dimensions of empathy across four subscales: Perspective Taking (PT), Fantasy (FS), Empathic Concern (EC), and Personal Distress (PD). PT measures the tendency to adopt the perspective of others, FS assesses the ability to imaginatively transpose oneself into the feelings of fictional characters, EC evaluates sympathy and concern for others, and PD gauges personal anxiety and concentration.

### Images acquisition and preprocessing

Imaging data were acquired on a 3-T MRI scanner (GE DISCOVERY MR750), with T1-weighted anatomical images were collected using a three-dimensional fast spoiled gradient-echo (3D FSPGR) sequence, and the scanning parameters were as follows: slices = 152; TR = 6.008 ms; TE = 1.984 ms; FOV = 256 × 256 mm2; FA = 9°; matrix = 256 × 256; slice thickness = 1 mm (no gap). The FreeSurfer 7.2.0 standard recon_all pipeline (https://surfer.nmr.mgh.harvard.edu/fswiki/recon-all) was used for processing and estimating the T1 imaging data^57^including motion correction, intensity normalization, Talairach transformation, and skull stripping. Subsequent steps involved segmentation and nonlinear alignment of white and gray matter structures, followed by cortical surface reconstruction and curvature computation.

### MIND structural similarity of brain regions

Morphometric analyses have advanced from region-wise averages to distribution-based descriptors. Morphometric similarity networks (MSNs) integrate multiple cortical features into biologically meaningful connectomes, yet they compress vertex-level information to regional means and assume homoscedasticity across features, thereby obscuring fine-scale cortical architecture^58^. Microstructural profile covariance (MPC) improves spatial precision by correlating depth-wise intensity gradients, but its reliance on high-resolution multimodal imaging and its omission of within-layer feature distributions can mask subtler effects^59^. Morphometric inverse network divergence (MIND) addresses these limitations by preserving the full multivariate distribution of voxel-wise morphometric features within each parcel. Specifically, MIND captures the joint distribution of cortical thickness(CT), mean curvature(MC), sulcal depth(SD), surface area(SA) and grey-matter volume(GMV), retaining intra-regional heterogeneity and cross-feature dependencies. This design confers unique sensitivity to non-Gaussian, spatially localized and potentially non-linear structural alterations—properties that are essential for detecting the subtle plastic changes induced by intensive training.

The cortical surface were first parcellated into 210 cortical regions based on Human Brainnetome Atlas (BN)^60^with each region corresponding to a distinct anatomical label. Using FreeSurfer, we mapped the standard BN atlas onto each participant’s cortical surface to achieve a customized parcellation. MIND were applied to further assess structural similarity between 210 cortical regions. This approach integrates five cortical features to estimate the similarity between brain regions. For each cortical region, a multidimensional distribution of multiple structural indicators (CT, MC, SD, SA and GMV) was established. The k-nearest neighbor algorithm^61^ was used to estimate the multivariate Kullback-Leibler (KL) divergence between pairs of regions, based on their Euclidean distances. MIND was then computed as the formula, in which $\:\widehat{\text{D}}({\text{P}}_{\text{a}\:},\:{\text{P}}_{\text{b})}$ represents a symmetric measure of multivariate KL divergence. According to formula, MIND values range from 0 to 1, with higher values meaning higher similarity of two regions. The details of calculation formulas and parameters for MIND can be found in Supplementary Material A.

$$\:\text{M}\text{I}\text{N}\text{D}(\text{a},\text{b})=\frac{1}{1+\widehat{\text{D}}({\text{P}}_{\text{a}\:},\:{\text{P}}_{\text{b})}}$$

### Statistical analysis

#### Behavioral data analysis

Behavioral data were first examined for normality using the Shapiro-Wilk test^62^. Age and years of education were not normally distributed (all *p* < 0.05); therefore, non-parametric Kruskal-Wallis test was used to assess group differences^63^. In contrast, all subscale and total scores of IRI demonstrated normal distributions (all *p* > 0.05), allowing for the use of parametric one-way analysis of variance (ANOVA) to evaluate group differences. All behavioral analysis were conducted using SPSS 26.0(IBM, Somers, USA), with a significance threshold set at *p* = 0.05.

#### MIND data analysis

For MIND analysis, each participant’s data yielded a 210 × 210 similarity matrix. Two complementary statistical approaches were used to analyze these matrices. First, we averaged each row of the matrix to calculate the mean MIND value for each brain region, reflecting its average similarity with the entire cortex. Second, we directly analyzed the full matrices to quantify pairwise similarity differences between regions. Group differences in both analysis were assessed with permutation tests with 10,000 iterations using Matlab2020b to evaluate the significance of both brain region MIND values and structural connectivity MIND values. False discovery rate (FDR) with a threshold of *p* < 0.05 was used for correcting for multiple comparisons.

Finally, partial correlation analyses were conducted to explore the relationship between MIND values and empathetic traits, as measured by the IRI. These analyses focused on brain regions showing significant group differences and controlled for sex, age, and years of education as covariates. Moreover, we also include the effect of years of training within dancers and musicians. All statistical procedures for MIND analysis were performed using MATLAB 2020b(The MathWorks, Inc). Brain network visualization was performed using BrainNet Viewer version 1.7 ^64^, a tool designed for the visualization of brain networks.

## Results

### Behavioral information

A total of 89 participants were included in this research, including 25 dancers, 24 musicians, and 40 healthy controls. There were no significant differences in age, gender, and educational years (Table 1). Descriptive statistics for empathetic traits are presented in Table 2. No significant group differences were found in subscale or total score of IRI. We also report the effect size of partial eta square and the 95% confidence interval(CI). Our results indicate that the EC and PD subscales exhibited relatively small effect sizes, whereas the PT, FS, and total IRI scores demonstrated moderate effect sizes^65^. The CI of IRI scales can be found in Supplementary Material B.

**Table 1:** Demographic information.

	Dancer group (*N* = 25)	Musician group (*N* = 24)	Health Control (*N* = 40)	*P* value
Gender (male/female)	8/17	15/9	20/20	0.09^a^
Age	20.04 ± 2.49	19.92 ± 1.44	20.51 ± 1.55	0.18^b^
Education years	13.76 ± 2.33	13.83 ± 1.63	14.69 ± 1.44	0.07^b^
Training years	9.46 ± 4.99	12.36 ± 3.50	-	-

*a: chi-square test; b: Kruskal-Wallis test.*

**Table 2:** IRI scale information.

		Dancer Group (*N* = 16)	Musician Group (*N* = 15)	Health Control (*N* = 20)	F value	*P* value
subscales						
	PT	18.75 ± 2.89	17.93 ± 4.77	20.55 ± 2.93	2.52	0.09
	FS	15.06 ± 3.49	17.13 ± 4.41	17.90 ± 4.76	2.01	0.15
	EC	19.00 ± 3.62	20.73 ± 4.54	20.45 ± 3.55	0.92	0.41
	PD	14.00 ± 2.60	15.00 ± 4.36	15.65 ± 3.70	0.93	0.40
Total score						
	IRI total	66.81 ± 6.70	70.80 ± 10.65	74.55 ± 10.81	2.86	0.07

### MIND result

**Fig. 1:**
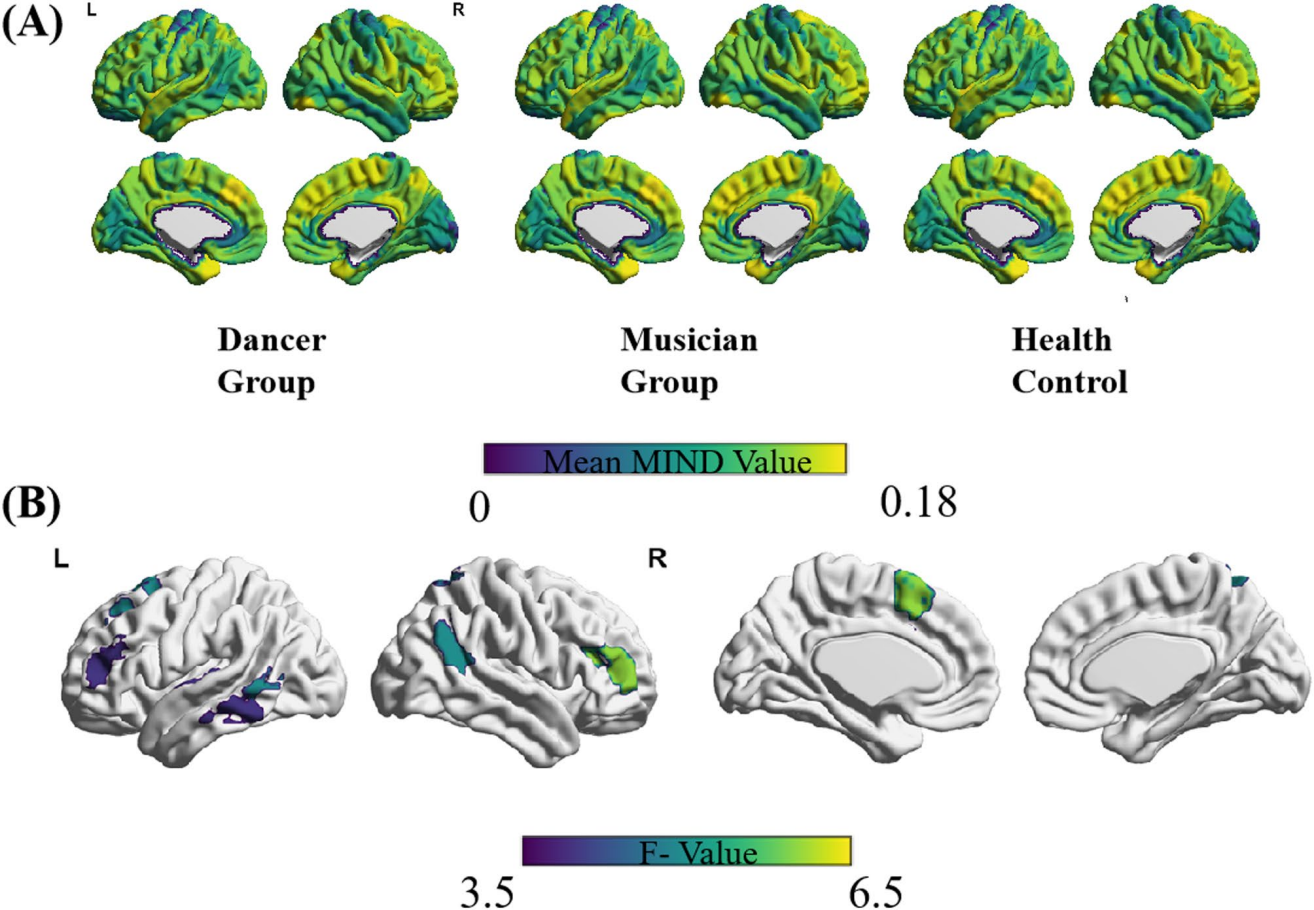
(**A**) Brain map of mean MIND values in three groups; (**B**) Brain regions showing significant differences among three groups.

**Fig. 2:**
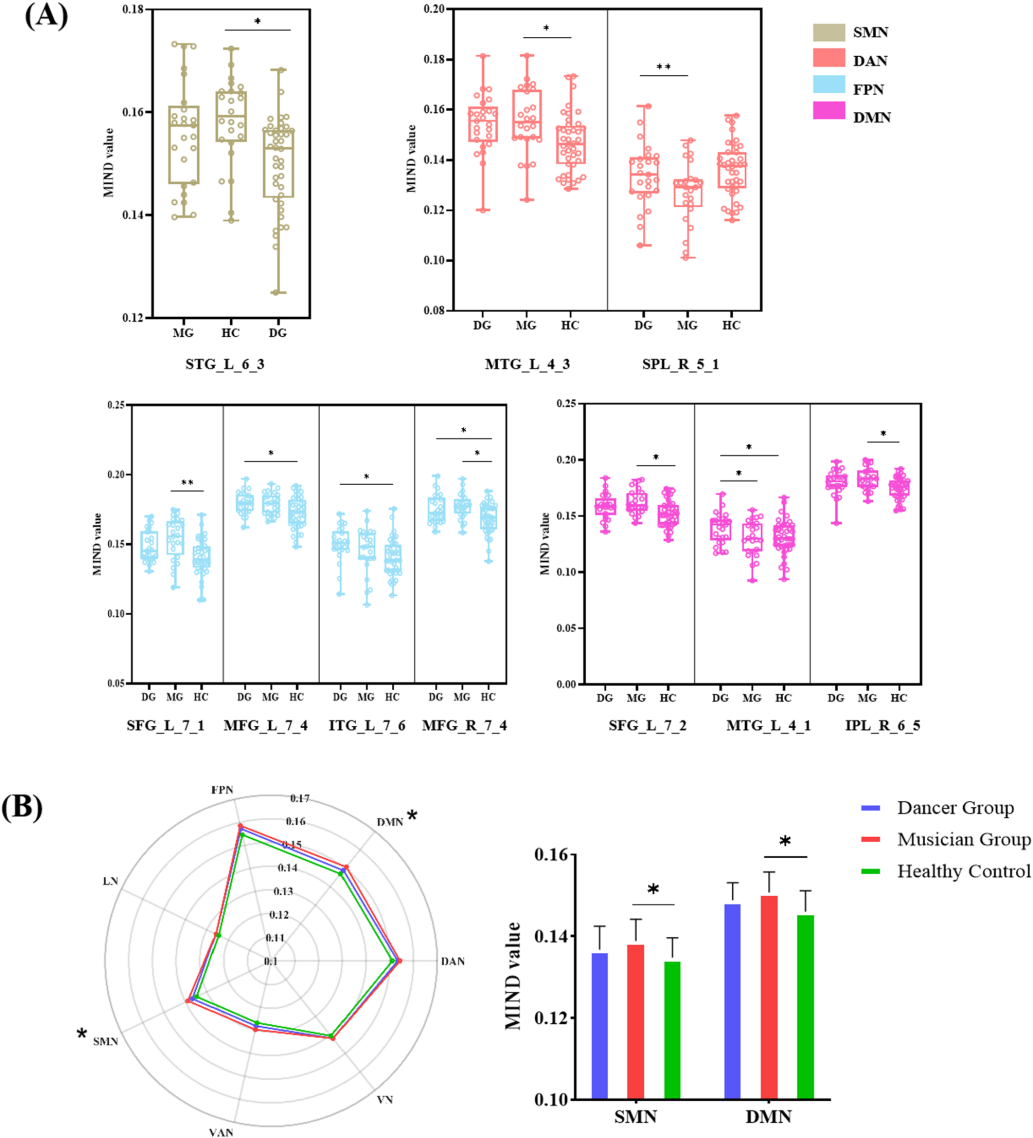
(**A**) Different brain regions in yeo - seven network; (**B**) Radar charts illustrating group differences in the yeo - seven networks MIND values. (*p < 0.05;**p < 0.01;***p < 0.001)

Group differences in MIND values were analyzed using permutation test. Table 3; Figs. 1 and 2 summarize the cortical regions showing significant differences among the three groups. These regions included left Superior temporal gyrus(STG), left Middle temporal gyrus (MTG), left Superior Frontal gyrus (SFG), right Middle frontal gyrus(MFG), left Inferior parietal lobule (IPL), right Infereior temporal gyrus (ITG), right superior parietal lobule(SPL), (Fig. 2.A). Most of the differential brain regions are located in the Frontal-parietal Network(FPN), Default Mode Network(DMN), and Dorsal Attention Network(DAN), followed by the Somatomotor Network(SMN). In addition, significant difference of MIND value can be found in DMN(*p* = 0.0203) and SMN(*p* = 0.0157), with the significant increased MIND in musician group compared with their health controls(Fig. 2.B).

**Table 3:** Mean MIND value.

		Dancers Group (*N* = 26)	Musicians Group (*N* = 24)	Controls Group (*N* = 40)	F value	FDR-corrected *P* value
Somatomotor Network (SMN)						
	STG_L_6_3	0.16 ± 0.01	0.16 ± 0.01	0.15 ± 0.01	4.20	2.79E-02
Dorsal Attention Network (DAN)						
	MTG_L_4_3	0.15 ± 0.01	0.16 ± 0.01	0.15 ± 0.01	5.28	2.24E-03
	SPL_R_5_1	0.13 ± 0.01	0.13 ± 0.01	0.14 ± 0.01	4.53	1.61E-02
Frontoparietal Network (FPN)						
	SFG_L_7_1	0.15 ± 0.01	0.15 ± 0.02	0.14 ± 0.13	6.45	2.49E-05
	MFG_L_7_4	0.18 ± 0.01	0.18 ± 0.01	0.17 ± 0.01	3.99	1.05E-04
	ITG_L_7_6	0.15 ± 0.01	0.15 ± 0.17	0.14 ± 0.13	4.31	2.79E-02
	MFG_R_7_4	0.18 ± 0.01	0.18 ± 0.01	0.17 ± 0.01	5.95	1.05E-04
Default Mode Network (DMN)						
	SFG_L_7_2	0.16 ± 0.01	0.14 ± 0.01	0.15 ± 0.01	5.00	3.62E-03
	MTG_L_4_1	0.14 ± 0.01	0.13 ± 0.02	0.13 ± 0.02	4.09	3.88E-02
	IPL_R_6_5	0.18 ± 0.01	0.18 ± 0.01	0.18 ± 0.01	4.54	1.46E-02

*Note: All brain region labels follow the Brainnetome (BN) Atlas nomenclature. For example*,* “STG_L_6_3” refers to the left superior temporal gyrus*,* subregion 6_3*,* as defined by the BN Atlas. MTG*,* middle temporal gyrus; SPL*,* superior parietal lobule; SFG*,* superior frontal gyrus; MFG*,* middle frontal gyrus; ITG*,* inferior temporal gyrus; IPL*,* inferior parietal lobule.*

**Fig. 3:**
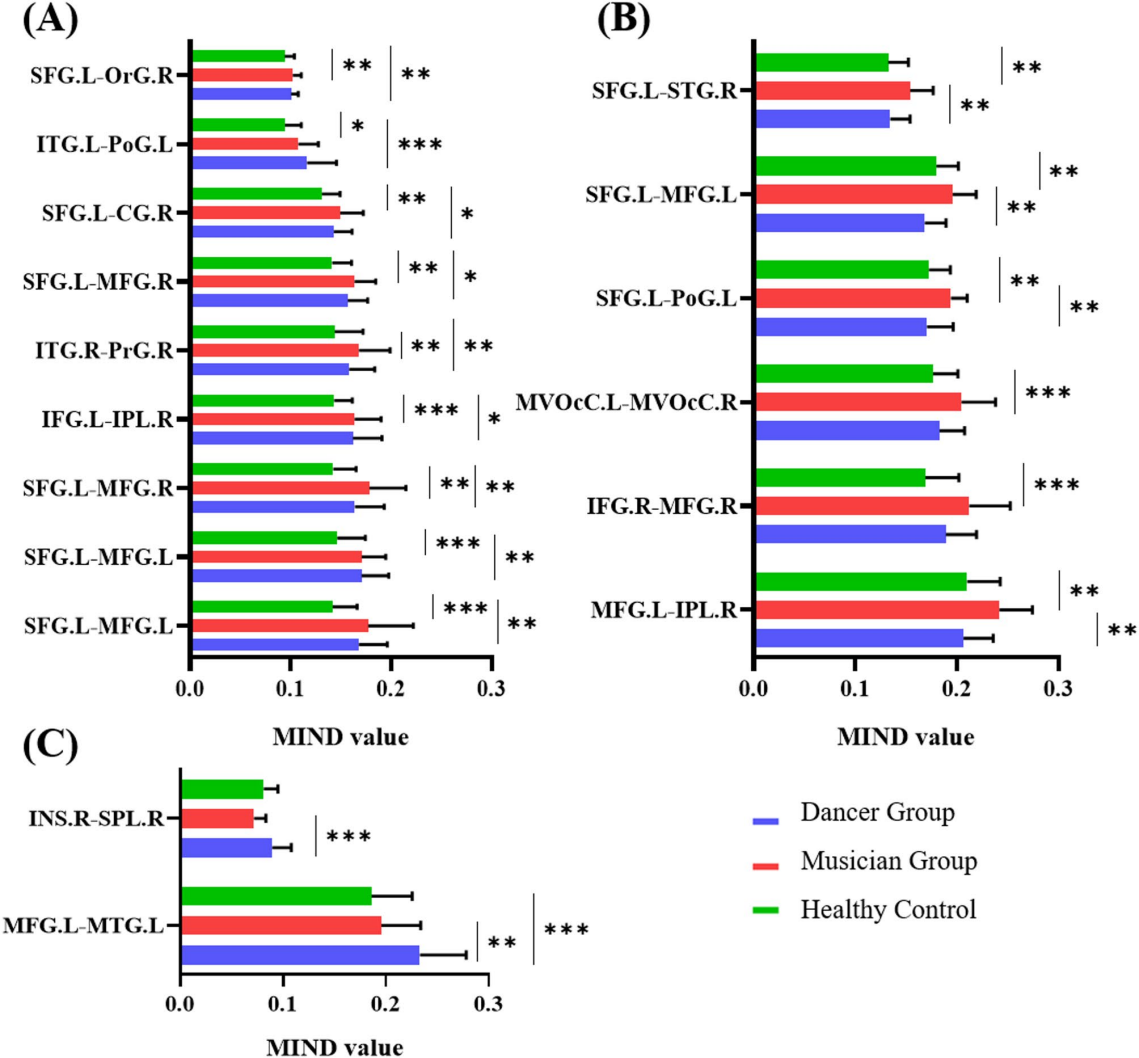
Significant different structural connectivity. (*p < 0.05, **p < 0.01, ***p < 0.001)

**Fig. 4:**
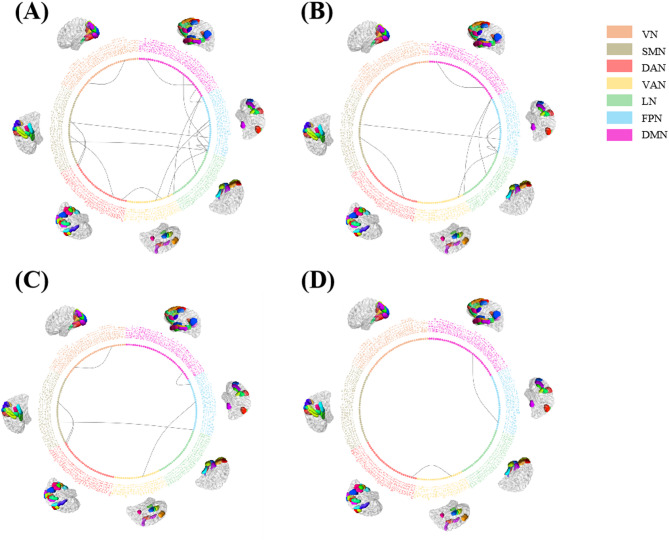
(**A**) All difference showed significant connectivity in Yeo seven network; (**B**) commonlity displayed the both musicians and dancers showed significant higher that health control; (**C**) specific MIND patterns in musician; and (**D**)specific MIND patterns in dancer.

Significant differences in MIND values for structural connectivity were also observed between the three groups. Table 4; Figs. 3 and 4 displayed specific information. The related results can also be found in Supplementary Material C. We identified three distinct patterns of connectivity.

**Table 4:** Structural connectivity of MIND.

Brain regions	Dancers Group (*N* = 26)	Musicians Group (*N* = 24)	Controls Group (*N* = 40)	F value	FDR - corrected *P* value
SFG_L_7_1 & MFG_R_7_4	0.164 ± 0.029	0.178 ± 0.036	0.141 ± 0.023	10.783	< 0.001
IFG_R_6_6 & MFG_R_7_4	0.189 ± 0.030	0.212 ± 0.040	0.167 ± 0.031	10.244	< 0.001
SFG_L_7_1 & MFG_R_7_2	0.157 ± 0.020	0.164 ± 0.021	0.141 ± 0.020	9.505	< 0.001
IFG_L_6_4 & IPL_R_6_5	0.162 ± 0.029	0.164 ± 0.026	0.143 ± 0.018	9.406	< 0.001
MFG_L_6_4 & IPL_R_6_4	0.206 ± 0.029	0.242 ± 0.032	0.210 ± 0.033	8.688	< 0.001
SFG_L_7_1 & MFG_L_7_3	0.168 ± 0.028	0.177 ± 0.045	0.140 ± 0.021	8.116	< 0.001
SFG_L_7_4 & PoG_L_4_2	0.170 ± 0.026	0.194 ± 0.016	0.172 ± 0.021	8.089	< 0.001
SFG_L_7_2 & MFG_L_7_7	0.171 ± 0.027	0.171 ± 0.024	0.146 ± 0.028	7.924	< 0.001
SFG_L_7_1 & STG_R_6_2	0.134 ± 0.020	0.154 ± 0.023	0.132 ± 0.020	7.920	< 0.001
MFG_L_7_6 & PoG_L_4_2	0.169 ± 0.021	0.196 ± 0.023	0.180 ± 0.022	7.845	< 0.001
SFG_L_7_1& CG_R_7_3	0.143 ± 0.018	0.149 ± 0.023	0.131 ± 0.018	7.836	< 0.001
MVOcC_L_5_5 & CG_R_7_2	0.096 ± 0.011	0.109 ± 0.010	0.100 ± 0.011	7.809	< 0.001
MFG_L_7_4 & MTG_L_4_4	0.233 ± 0.045	0.196 ± 0.038	0.186 ± 0.040	7.778	< 0.001
MVOcC_L_5_2 & MVOcC_R_5_5	0.183 ± 0.024	0.204 ± 0.034	0.176 ± 0.025	7.751	< 0.001
ITG_R_7_5 & PrG_R_6_4	0.158 ± 0.025	0.168 ± 0.031	0.144 ± 0.028	7.710	< 0.001
INS_R_7_5 & SPL_R_5_5	0.090 ± 0.018	0.072 ± 0.011	0.082 ± 0.014	7.642	< 0.001
ITG_L_7_6 & PoG_L_4_4	0.116 ± 0.029	0.108 ± 0.020	0.094 ± 0.016	7.620	< 0.001
SFG_L_7_2 & OrG_R_6_4	0.100 ± 0.007	0.102 ± 0.008	0.094 ± 0.010	7.552	< 0.001

*Note: All brain region labels follow the Brainnetome (BN) Atlas nomenclature. For example*,* “SFG_L_7_1” refers to the left superior temporal gyrus*,* subregion 7_1*,* as defined by the BN Atlas. MFG*,* middle frontal gyrus; IFG*,* inferior frontal gyrus; IPL*,* inferior parietal lobule; PoG*,* postcentral Gyrus; STG*,* superior temporal gyrus; CG*,* cingulate gyrus; MVOcC*,* medioventral occipital cortex; ITG*,* inferior temporal gyrus; PrG*,* precentral gyrus; INS*,* insular gyrus; SPL*,* superior parietal lobule; OrG*,* orbital gyrus.*

1. **Common enhancement pattern**: both musicians and dancers exhibited significantly higher that HC with no significant difference between dancers and musicians. These connectivity including right SFG and left Orbital Gyrus(OrG), left ITG and right Postcentral Gyrus(PoG), left SFG and left CG, left SFG and right MFG, right ITG and right Precentral Gyrus(PrG), left IFG and right IPL, left SFG and both left and right MFG.
2. **Musician-specific enhancement**: Musicians showed the highest MIND values and showed significant difference between HC or dancers, which contains connectivity between left MedioVentral occipital cortex (MVOcC) and right IPL, right SFG and right SFG, MVOcC and right CG, left SFG and right STG, left SFG and left MFG, left SFG and left PoG, left MVOcC and right MVOcC, right IFG and right MFG, left MFG and right IPL.
3. **Dancer-specific enhancement**: Dancers exhibited significantly higher MIND values compared to musicians and healthy controls in two specific connections: between the right INS and right SPL, and between the left MFG and left MTG.

### MIND value correlate with empathy

**Fig. 5:**
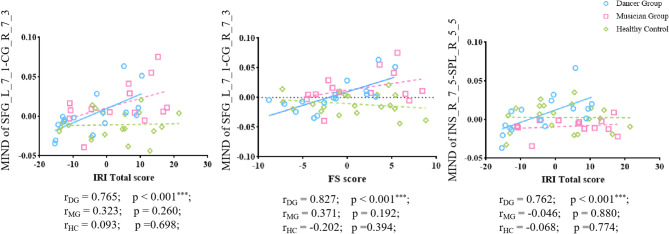
The positive correlation between MIND value and IRI scales and FS scale in dancer group.(*p < 0.05, **p < 0.01, ***p < 0.001).

Finally, we examined the association between MIND connectivity and empathetic traits. In the dancer group, the MIND value between the _left SFG_7_1 and right CG_7_3 positively correlated with the IRI total score (*r* = 0.765, *p* < 0.001) and the FS score (*r* = 0.827, *p* < 0.001) in dancer group. Similarly, connectivity between the right INS (INS_6_3) and right SPL (SPL_5_5) was positively correlated with the IRI total score (*r* = 0.762, *p* < 0.001) (Fig. 5).

## Discussion

In this study, we applied a novel approach based on structural similarity of brain regions to investigate training-induced plasticity associated with dance and music. Our findings reveal not only shared neural adaptations between the two art forms, but also distinct, modality-specific changes. Notably, these structural variations were linked to higher-order socio-emotional capacities, particularly empathy, which offering new insights into the neural mechanisms underlying art therapies.

### Shared MIND patterns of SMN and DMN in dance and music training

Myelination may be a key mechanism underlying the elevated MIND values observed in this study. Long-term and repetitive professional training is known to promote myelination in task-relevant brain regions, thereby enhancing neural conduction efficiency and increasing structural similarity^66–67^. For instance, musicians with absolute pitch exhibit greater cortical myelination in auditory-related regions of the temporal lobe^68^. A 12-year longitudinal study further demonstrated sustained increases in white matter myelination as a result of continuous musical training^68^. In addition, both high-intensity aerobic exercise and dance-based interventions have been shown to enhance myelination, with particularly pronounced effects observed in older adults^69–70^. These findings suggest that music and dance training may promote activity-dependent myelination in relevant brain networks.

In the present study, elevated MIND values were identified in both the sensorimotor network (SMN) and the default mode network (DMN) in dancers and musicians, potentially reflecting enhanced intra-network integration associated with long-term training. This pattern is consistent with previous findings using network morphometric similarity analysis, in which chess masters exhibited significantly greater morphometric similarity within the SMN and central executive network compared to amateurs^71^. The SMN plays a central role in motor planning and execution^72^whereas the DMN is implicated in self-referential processing, introspection, and episodic memory retrieval^73,74^. Musical performance typically involves fine motor control^76–78^particularly of the fingers, while dance training emphasizes large-scale, whole-body movements^79–81^. As immersive art forms, both music and dance also elicit strong emotional and introspective engagement, thereby necessitating sustained DMN involvement^82–86^. Prior research has demonstrated increased DMN activation and functional connectivity during music listening and dance observation^87–89^.

In addition to myelination, synaptic pruning may also contribute to increased structural similarity^90^. As functional specialization progresses, dendritic structures undergo selective elimination, thereby enhancing neural efficiency and refining motor and cognitive processes^91–92^. For example, decreased GMV and WMV have been observed in the SMN area including left premotor cortex, SMA^93–94^ in dancers, possibly reflecting greater neural efficiency.

### Distinct MIND patterns in dancers and musicians

Beyond these shared patterns, we identified distinct MIND profiles in dancers and musicians. Specifically, musicians exhibited the highest MIND values in the left STG area and showed increased connectivity between left SFG and right STG, indicating that STG as a key region in musicians. Located adjacent to Heschl’s gyrus, the STG is critical for auditory perception and higher-order auditory processing^95 ,96^, and plays an essential role in music perception^97–99^. Previous studies have demonstrated enhanced activation in the anterior STG and Heschl’s gyrus during music listening and imagination in musicians^100–101^. The increased similarity between the SFG and STG observed here may indicate that music training enhances auditory processing and its integration with cognitive control regions.

Dancer demonstrated higher MIND values in left MTG and greater connectivity between left MFG and MTG. The MTG is a key node of the DMN and is implicated in audiovisual integration, particularly during dance movement^102,103^. Prior studies have reported increased cortical thickness in the MTG of musicians and dancers^81^ and improved motor performance following MTG stimulation during dance tasks^104^. Additionally, functional near-infrared spectroscopy (fNIRS) research has linked MTG activity to the successful integration of visual and rhythmic cues^105^. Given that dance requires continuous integration of auditory rhythms and precise motor execution, the elevated MIND values in the MTG likely reflect long-term multimodal integration demands in dancers^106^.

### Distinct empathy mechanism induced by dance training

Importantly, we found a specific association between MIND values and empathetic traits in the dancer group. In particular, MIND similarity between the left SFG and right CG, as well as between the right SPL and right insula (INS), was positively correlated with total IRI scores. Additionally, connectivity between the left SFG and right CG was positively associated with the Fantasy (FS) subscale of the IRI. These findings suggest that dance training may specifically enhance the capacity for empathetic imagination.

The cingulate gyrus and insula are key components of the limbic system and are implicated in higher-order cognitive, emotional, and memory functions. Previous studies have reported increases in the volume of the cingulate cortex and insula following six months of dance intervention^107^, as well as enhanced resting-state FC and synchronized neural activity between the insula, dorsomedial frontal cortex, and other related regions in professional dancers^47–48,108^. The concept of kinesthetic empathy proposes that dance enables individuals to infer others’ emotional states through the simulation of movemen^28–29^, a process potentially mediated by dance imagery. As an emotionally expressive art form, dance incorporates various forms of auditory and kinesthetic imagery^109^. These mental images are consciously created and reflect the dancer’s imaginative emotional state^110^, which can enhance emotional expression and performance preparation^111–113^. Additionally, many dance forms, such as ballroom dancing, rely heavily on nonverbal interpersonal communication^114^. Dancers must accurately perceive and interpret their partners’ emotional states to achieve synchronization and effective collaboration^45,^^115–117^. Together, these elements of dance practice promote a rich imaginative experience that may strengthen empathetic fantasy.

In contrast, no significant correlations between MIND values and empathetic traits were observed in the musician group. This suggests that, while both music and dance training influence cortical similarity, the embodied, social, and expressive nature of dance may uniquely enhance the cognitive and neural mechanisms underlying empathetic imagination.

## Limitation

Despite the interesting findings of this study, several limitations should be considered. Firstly, while musicians demonstrated a trend towards a positive correlation with empathy, this relationship did not reach statistical significance. This may be attributed to the limited empathy data involved in the current study, which could have impacted the detection of subtle associations. Besides, this study relied on a self-report measure of empathy (IRI), which may be influenced by social desirability and individual differences in introspection, potentially contributing to ceiling effects. Future studies should incorporate behavioral or performance-based measures to provide a more objective assessment of empathic ability. Future studies should aim to incorporate more extensive data and varied empathy measures to better explore the potential link between musical training and empathy.

While we recorded years of training as part of participant inclusion criteria, this measure does not capture training intensity or physical fitness. Objective indicators such as VO₂max or resting heart rate were not assessed and could influence brain plasticity, particularly in physically demanding disciplines like dance. As a result, we cannot fully separate the effects of general fitness from those of domain-specific training. Future studies should include standardized measures of training load and fitness to better isolate expertise-related neural adaptations.Finally, the cross-sectional nature of this study restricts the ability to infer causality between long-term dance training and empathy development. A longitudinal design would allow for a more comprehensive understanding of how prolonged exposure to dance or music training might influence empathetic abilities over time. Moreover, future research could benefit from experimental designs that manipulate the type and duration of training to establish stronger causal relationships.

## Conclusion

Above all, our results complement the structural evidence that long-term music and dance training can affect brain regions similarities. They have both similarities and distinctions. While musician result more auditory similarity, dancers showed more resemblance in audiovisual integration region. In addition, the effects dance training on brain plasticity correlate with the overall empathic ability, and dance training specifically affects the empathetic imagery of dancers.

## Supplementary Information

Below is the link to the electronic supplementary material.

Supplementary Material 1

## Data Availability

The datasets used and analyzed during the current study are available from the corresponding author on reasonable request.
