# Supplementary material for: Shared and distinct patterns of cortical morphometric inverse divergence and their association with empathy in dancers and musicians
Source: Sci Rep. 2025 Aug 5;15:28572. doi: 10.1038/s41598-025-13416-2 (PMC12325637; doi:10.1038/s41598-025-13416-2)
Supplement: Supplementary file 1 — Supplementary Material 1 [file 41598_2025_13416_MOESM1_ESM.docx]

Supplementary Material A

According to the processing flow of free surfer, five features of each brain region were extracted based on T1-weighted images, including GMV, sulcus depth, cortical thickness, mean curvature and surface area. Subsequently, morphological metrics were obtained for all vertices of each brain region. For a given pair of regions A and B, their vertex sets are denoted as Va and Vb, and the multivariate probability density distribution functions of their vertices are $P_{a}$and $P_{b}$. In this study, firstly, the KL scatter estimated using the K nearest neighbour scatter approximation estimation method, denoted as $\hat{D_{KL}}\left( P_{a}\parallel P_{b} \right)$ , as shown in Equation 1.

$\hat{D_{KL}}\left( P_{a}\parallel P_{b} \right)= -\frac{d}{n}\sum_{i=1}^{n} log\frac{r_{k}(x_{i)}}{s_{k}(x_{i)}}+log\frac{m}{n-1}$ (Equation 1)

In the formula, d denotes the number of morphological features; n and m represent the number of vertices in the vertex sets $V_{a}$ and $V_{b}$, respectively. $x_{i}$ represents the ith vertex in Va, and $r_{k}(x_{i)}$ and $s_{k}(x_{i)}$ represent the Euclidean distances from vertex $x_{i}$ to the kth nearest-neighbour vertex in Va (excluding the $x_{i}$ vertex itself) and $V_{a}$, respectively. In this study, d=5, k=1.

Due to the asymmetric nature of KL dispersion, a symmetric version of this metric was used. As shown in Equation 2.

$\hat{D}\left( P_{a}\parallel P_{b} \right)= \hat{D_{KL}}\left( P_{a}\parallel P_{b} \right)+\hat{D_{KL}}\left( P_{b}\parallel P_{a} \right)$ (Equation 2)

Considering the possibility that the estimate of the KL scatter may be negative, although the probability is extremely low single does exist, the minimum value of the estimate is set to 0. The symmetry index of the KL scatter is subsequently given as Equation 3.

$\hat{D}\left( P_{a}\parallel P_{b} \right)= max(\hat{D_{KL}}\left( P_{a}\parallel P_{b} \right),0)+max(\hat{D_{KL}}\left( P_{b}\parallel P_{a} \right),0)$ (Equation 3)

Finally, the MIND value can be calculated as:

$MIND(a,b)=\frac{1}{1+\hat{D}(P_{a}, P_{b)}}$ (Equation 4)

Supplementary Material B

**Table 1 IRI scale 95% Confidence Interval(CI)**

|  |  | Dancer Group  [lower limit, upper limit] | Musician Group  [lower limit, upper limit] | Health Control  [lower limit, upper limit] |
| --- | --- | --- | --- | --- |
| subscales |  |  |  |  |
|  | PT | [17.21, 20.29] | [15.29, 20.58] | [19.18, 21.92] |
|  | FS | [13.20, 16.92] | [14.69, 19.57] | [15.67, 20.13] |
|  | EC | [17.07, 20.93] | [18.22, 23.25] | [18.79, 22.11] |
|  | PD | [12.64, 15.36] | [12.59, 17.41] | [13.92, 17.38] |
| Total score |  |  |  |  |
|  | IRI | [63.24, 70.38] | [64.90, 76.60] | [69.49, 79.61] |

Supplementary Material C

Figure S.1 illustrates the position of the observed F-values for MIND-based structural connectivity within the null distribution generated by 10,000 permutation tests. The results indicate that the observed F-values fall within the extreme tail of the null distribution, supporting their statistical robustness.


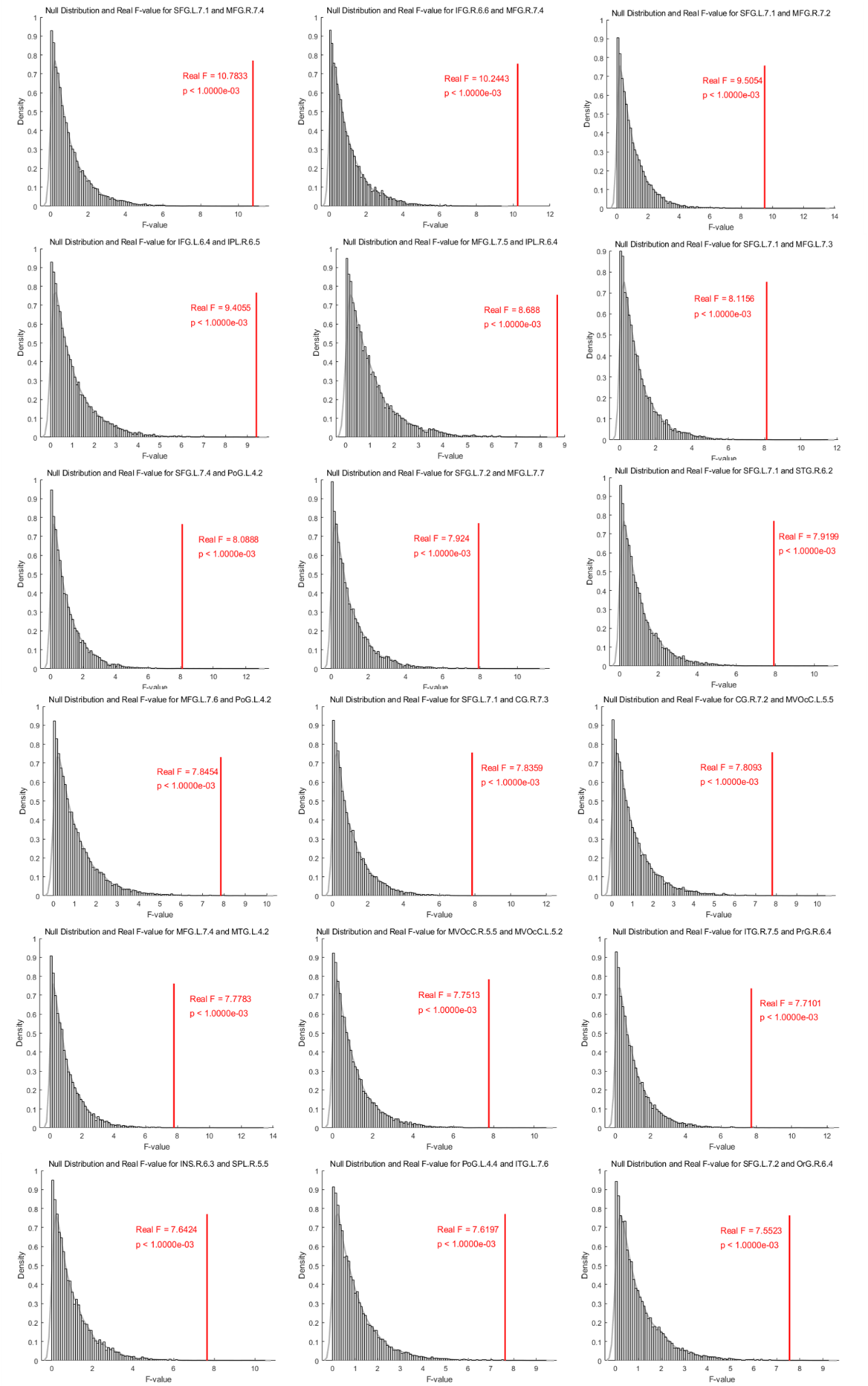


Figure S.1 Permutation Test Results for MIND-Based Structural Connectivity
